# Early Molecular Response to Imatinib First-Line Therapy and Predictive Factors of Poor Outcomes for Chronic Myeloid Leukemia Patients in Côte d'Ivoire

**DOI:** 10.1155/2024/4576455

**Published:** 2024-12-11

**Authors:** Kouassi Gustave Koffi, Sara Akou Bognini, Dohoma Alexis Silué, Ismael Kamara, Ines Kouakou, Emeraude N'dhatz, Boidy Kouakou, Danho Clotaire Nanho, David Tea Okou

**Affiliations:** ^1^Department of Hematology Teaching Hospital, Centre Hospitalier Universitaire de Yopougon, 21 BP 632, Abidjan 21, Ivory Coast; ^2^Department of Genetics, Clinic Saint George, Abidjan, Ivory Coast; ^3^Department of Health Public Hospital, Institut National de Sante Publique (INSP), Abidjan, Ivory Coast; ^4^Department of Pediatrics, Emory University School of Medicine, Atlanta, Georgia, USA

**Keywords:** Africa, chronic myeloid leukemia, imatinib, molecular response

## Abstract

**Objective:** The present study aimed to evaluate for the first time, the early molecular response (EMR) to imatinib at 3 months for patients with chronic myeloid leukemia and to determine the predictive factors that influence poor outcome and response.

**Methods:** 60 newly diagnosed CML patients were enrolled from May 2018 to June 2023. They received imatinib and prospectively underwent a molecular evaluation. Their EMR was assessed using a RT-qPCR method and expressed as the *BCR::ABL1* IS transcript level at 3 months. Potential factors impacting the EMR were identified using the Cox proportional hazard regression models. The effects of an EMR on the cumulative incidence of a deep molecular response (DMR) were also evaluated.

**Results:** Out of the 60 CML patients recruited, 29 (48%) achieved an optimal response with TKI therapy after 3 months. The cumulative rate of molecular response was 16 (36%) for a major molecular response (MMR), 10 (23%) for MR4, 8 (18%) for MR4.5, and 6 (14%) for MR5, while 4 (9%) showed indetectable transcript. In addition, as 26 (90%) of patients with optimal response at 3 months showed a DMR, we determined that an optimal response to TKI at 3 months was significantly correlated with a DMR. We also identified through multivariate analysis that seven independent risk factors significantly influenced an EMR to TKI. These factors included male, late diagnosis, advanced performance status, the presence of splenomegaly, high-ELTS risk groups, a *BCR::ABL1* domain mutation, and complete hematologic response after more than 30 days.

**Conclusion:** Our study demonstrates that an EMR at 3 months has a predictive value for a DMR. In addition, a MMR and a DMR can be predicted using a combination of parameters that either have a significant impact on the optimal response, or that can serve as prognostic indicators for molecular response, especially in low-income countries, where molecular assessment and monitoring are not available or possible.

## 1. Introduction

Chronic myeloid leukemia (CML) is a clonal myeloproliferative disorder of a pluripotent stem cell one first described by John Hughes Bennett in 1845. It was the first malignancy that had a specific chromosomal abnormality uniquely linked to it after the discovery of a minute chromosome now known as the Philadelphia (Ph) chromosome, later defined to result from a t (9; 22) reciprocal chromosomal translocation [1, 2]. This translocation results in the production of *BCR::ABL1* oncoprotein fusion with tyrosine kinase activity. Therefore, the tyrosine kinase inhibitor (TKI) therapy has been the main targeted treatment for CML. The management of CML has improved over the last decade and has turned CML into a chronic manageable disease from a near-fatal condition. Although the natural history of CML was forever changed with the arrival of TKI therapy, it remains a burden in resource-poor African countries. The majority of CML patients in the world live in low- and middle-income countries and have a very limited access to medical care and to diagnostic tools and therapies. However, within the past 10 years, the promising TKI therapy became available for the treatment of African patients with CML, with the availability of free imatinib mesylate donated by the Glivec International Patient–Assistance Program (GIPAP). This drug has become the first-line therapy for CML in sub-Saharan Africa, including Côte d'Ivoire, by providing a great deal of relief to patients. Accordingly, the prognosis of CML has changed from a fatal disease to a disease that is compatible with a normal lifespan. Detection of peripheral blood (PB) *BCR::ABL1* mRNA levels by quantitative reverse transcription polymerase chain reaction (RT-qPCR) is the most sensitive method for monitoring CML and the efficacy of TKIs. Following therapy, the quantitative measurements of residual disease by RT-qPCR of *BCR::ABL1* mRNA provide a measure of the molecular response and are highly correlated with outcome measurements. Monitoring the molecular response is necessary to identify suboptimal response to detect treatment failure early and to guide the decision to switch to alternative more efficacious therapies. This also means identifying patients who are resistant to TKI therapy. In case of failure or suboptimal response to imatinib, a mutation analysis of the *BCR::ABL1* kinase domain is recommended.

Achievement of a major molecular response (MMR) according to the International Scale (IS) puts the patient in a “safe harbor” zone where resistance to treatment and disease progression is unlikely. Furthermore, a deep molecular response (DMR) (a molecular response of four or lower) is one of the main criteria that reveals patients eligible to discontinue TKI therapy [3, 4]. In low- and middle-income countries of sub-Saharan Africa, diagnosis and monitoring of CML are not routinely performed because of limited resources and higher costs. We therefore opted for north–south subcontracting through the Cerba laboratory in Paris and also with some national private laboratories specialized in molecular cytogenetics by the GeneXpert system in order to fill the diagnostic gap of cytogenetics diagnosis of CML in our country.

The data on the assessment of residual disease and the molecular response to imatinib are scarce in Africa. The very few studies that have been conducted generally outline the suboptimal response seen in patients from resource-limited countries, as opposed to patients from more developed countries. [5]. Sub-Saharan African countries, including Côte d'Ivoire, have mostly reported studies on the treatment with TKIs but not on their outcomes. In Côte d'Ivoire, many gaps in knowledge and practice have been identified which may negatively impact CML management. This includes the assessment of residual disease and the monitoring of the molecular response to TKIs. Subsequently, this study aimed to report for the first time, data on the molecular response to imatinib and to assess the predictive factors for the poor outcome and response seen in patients after imatinib failure.

## 2. Materials and Methods

### 2.1. Patients and Inclusion Criteria

The clinical hematology department of the University Hospital Center of Yopougon (CHUY) in Abidjan, is the referral center for the management of hematology disorders in Cote d'Ivoire. We performed a prospective trial of imatinib mesylate on patients with newly diagnosed CML. All patients treated for CML at CHUY have been registered prospectively in our leukemia database, which was established in 2005 through the “Max Access Program” of the Max Foundation. The study cohort included CML patients with documented *BCR::ABL1* positive gene test, who were enrolled in the GIPAP from May 2018 to June 2023. The diagnosis of CML was confirmed prior to imatinib mesylate treatment by morphological examination of PB and by documentation of the presence of the *BCR::ABL1* fusion gene using quantitative real-time PCR (RT-qPCR). There is a financial burden on the patients associated with the higher cost of RT-qPCR tests. To help patients overcome that burden, the Max Foundation, through the GIPAP has assisted with a low-cost diagnostic approach [6]. This approach performs the quantification and detection of the *BCR::ABL1* fusion gene on samples collected on dry blood spot (DBS) absorbent filter paper. DBS preserves nucleic acid in the dried blood spots for subsequent laboratory tests. This approach is free to the patients, as part of the GIPAP and helps with the diagnosis and molecular follow-up of patients on TKI therapy in low-income countries. It also helps to guide therapeutic decisions in case of resistance or failure of first-line therapy with imatinib mesylate. Demographics, clinical and laboratory information, and data were collected at the time of diagnosis. A total of 60 newly diagnosed CML chronic patients who received imatinib treatment (400 mg/day) as a first-line therapy and underwent a cytogenetic evaluation were prospectively followed. Thirty of them who showed suboptimal response underwent testing for *BCR::ABL1 domain* mutation screening through the GIPAP access diagnosis. All of the patients provided written informed consent to participate in the studies. The complete details on the study population are presented in Table 1.

### 2.2. *BCR::ABL1* Quantification

For the detection and monitoring of the *BCR::ABL1* fusion gene, the GeneXpert platform and the calibrated Xpert *BCR::ABL1* Monitor (a cartridge-based automated assay) from Cepheid were used. PB samples of patients collected in EDTA tubes or spotted on absorbent filter paper (DBS) were used for the detection and monitoring of the *BCR::ABL1* fusion gene. The samples were analyzed using a 4-module GeneXpert platform system (Cepheid, Sunnyvale, CA, USA).

For the samples collected in EDTA tubes, the assay was performed on 200 μL of PB according to the manufacturer's instruction. In brief, the sample was placed in lysis buffer, vortexed, and incubated at room temperature for 10 min. Ethanol was added to precipitate the nucleic acids and the mix was placed into the Xpert *BCR::ABL1* Ultracartridge. The cartridge was inserted into the GeneXpert platform which analyzed the sample using preset cycling conditions. The results are reported on an IS, with the amount of *BCR::ABL1* presented as a percentage (*BCR::ABL1*/*ABL1*) aligned to the IS via an internal batch–controlled conversion factor. The significance of the *BCR::ABL1* transcript level is considered in the context of treatment and of subsequent *BCR::ABL1* testing.

For the samples spotted on the absorbent filter paper, 200 μL of PB was collected per card (50 μL per spot, on 4 spots). The cards were dried for 2 days on the laboratory bench, after which, they were sent to the Fred Hutch Cancer Research Center laboratory in Seattle. Subsequent to the extraction of RNA from the DBS, samples were analyzed on the GeneXpert platform as described above.

### 2.3. *BCR::ABL1* Kinase Domain Mutational Analysis

Thirty patients underwent *BCR::ABL1* kinase domain mutation screening using a sequencing method performed through a collaboration with Fred Hutch Cancer Research Center laboratory in Seattle (USA). In brief, RNA was extracted from a single spot (50 μL blood) of the absorbent filter paper. The *BCR::ABL1* fusion gene was amplified by a RT-PCR and its kinase domain was further amplified via a nested PCR. The *BCR::ABL1* kinase domain was then sequenced in the forward and reverse directions using Dye Terminator Chemistry, and the results were compared with a wild-type reference sequence to identify mutations.

### 2.4. Treatment With Imatinib

The patients were treated according to protocols approved by the institutional review board, and informed consent for the treatment was obtained in accordance with the Declaration of Helsinki. All patients received a daily 400 mg oral dose of imatinib. No concomitant chemotherapy was administered other than a short treatment of hydroxyurea when deemed necessary for those who needed it. The imatinib dosage was adjusted depending on the tolerance and response of the recipient. To remain fully compliant with the GIPAP, the CML patients were required to pick up their medication (box of imatinib) in the hospital setting and under medical supervision. Also, to receive their monthly supply of medication, the CML patients were required to bring and show the empty drug blister packs of their previously consumed supply. Whenever possible, the medication dose was maintained above 300 mg/day. However, when Grade 3/4 thrombocytopenia or Grade 3/4 neutropenia was observed, the medication dose was reduced. Patients with an accelerated and blast phase according to cytological and conventional cytogenetic were not included in our studies. Were also excluded were patients who were taking medications other than imatinib, and patients who were noncompliant with imatinib therapy.

### 2.5. Cytogenetic Evaluation and Therapeutic Molecular Response

The molecular response was assessed using RT-qPCR and expressed as the *BCR::ABL1*/*ABL1* ratio (IS). The level of *BCR::ABL1* mRNA (transcripts) was generally assessed every 3 months for the first year, and then every 6 months thereafter. The molecular response is defined as the magnitude of reduction of the *BCR::ABL1* transcripts compared to its original value of 100%. The molecular response was categorized into 3 groups according to the European LeukemiaNet (ELN) 2020 guidelines [7] as follows: (1) Optimal molecular response: at 3 months, *BCR::ABL1* ≤ 10%; at 6 months, *BCR::ABL1* ≤ 1%; and at 12 months, *BCR::ABL1* ≤ 0.1%. (2) Warning: at 3 months, *BCR::ABL1* > 10%; at 6 months, *BCR::ABL1* > 1%–10%; and at 12 months, *BCR::ABL1* > 0.1%–1%.(3) Failure: at 3 months, *BCR::ABL1* > 10% if confirmed within 1–3 months; at 6 months, *BCR::ABL1* > 10%; and at 12 months and anytime *BCR::ABL1 IS* > 1%. The achievement of a MMR (MMR ≤ 0.10% IS) and a DMR was evaluated. A molecular response of 4.0 (MR4) is defined as 0.0032%< *BCR::ABL*1^IS^ ≤ 0.01% (*ABL1* transcripts ≥ 10,000), a molecular response of 4.5 (MR4.5) as 0.001% < *BCR::ABL*1^IS^ ≤ 0.0032% (*ABL1* transcripts ≥ 32,000), and a molecular response of 5.0 (MR5.0) as *BCR::ABL1* ≤ 0.001% (*ABL1* transcripts ≥ 1,00,000). A DMR is defined as ≥ MR4.0 [8]. For all the patients followed, the responses achieved at any time point during the treatment were recorded and assessed.

### 2.6. Statistical Analysis

The SPSS software version 25.0 was used for statistical analysis. The qualitative variables were expressed as numbers and percentages and the quantitative variables were expressed as a mean with standard deviation. The Student's *t*-test was used to compare the means, while the comparison of percentages was made using Fisher's exact test. For statistical significance, a *p* value < 0.05 was considered. A multivariate analysis using a logistic regression model was carried out to determine the factors associated with an optimal response to TKI after 3 months of therapy. The odds ratio (OR) and confidence interval (CI) were used to determine an association between the exposure to imatinib and the outcomes. Given the size of the samples, the Yates continuity correction was applied to contingency tables containing the value “0” in order to make it possible to calculate the OR and its CI. The likelihood ratio test was used to assess the significance of the OR and their 95% CIs.

**Informed consent** was obtained from all the subjects and includes a provision that allow any participants to withdraw from the study at any point in time.

The study was approved by the institutional ethics committee and conducted according to the Declaration of Helsinki.

## 3. Results

### 3.1. Sociodemographic and Clinical Characteristics of Patients With CML

From May 2018 to June 2023, 140 patients with chronic phase–CML (CP–CML) were treated with imatinib. Only 60 patients underwent a molecular cytogenetic evaluation, 30 of which underwent mutational screening during follow-up. The clinical and biological characteristics of all patients are shown in Table 1. The median age of patients was 45 years (range: 17–75 years), with 34 and 26 patients being male and female, respectively. More than 60% of patients were at a poor socioeconomical level and had no insurance. The ECOG performance at diagnosis was 1 and 2 for the majority (77%) of patients. The median time to diagnosis was 1.5 months (0.3–7.5). The ELTS risk stratification group classified the patient into low-ELTS (47%), intermediate-ELTS (20%), and high-ELTS (33), respectively. The majority of patients (75%) had splenomegaly, while 13% had hepatomegaly. The mean total leukocyte count was 298.3 ± 210 × 10^9^/L with a range from 10.7 to 960 × 109/L. The mean hemoglobin was 9.6 ± 1.6 g/dL with a range from 6 to 15 g/dL. The mean platelet count was 588 ± 292 × G/L with a range from 144 to 1653 G/L. The median time from the diagnosis to the start of the imatinib treatment was 15 days (range: 5–90 days).

### 3.2. *BCR::ABL1* Ratio and Mutation Screening

The mean *BCR::ABL1* transcript percentage at diagnosis was 54% (range: 29–150). Of the 60 patients who were monitored in this study, only 30 underwent mutational screening of the kinase domain in order to evaluate the prognostic impact of the mutation profile. We identified 10 previously known *BCR::ABL1* mutations that are summarized in Table 2. We identified a single T315I mutation in 3 patients, while 2 patients carried a double mutation each, one with the T315I/M244V mutation and another with the G250E/E459K mutation.

### 3.3. Predictive Factors for Outcome and Molecular Response to Imatinib Treatment

The complete hematologic response (CHR) rate in our study group was 83%, which was achieved within a median time of 2 months (range: 0.6–6 months). Out of the 60 CML patients monitored, 29 (48%) achieved an optimal response with TKI therapy after 3 months by RT-qPCR measurement (Table 3). At the end of the trial, different molecular responses were seen in 44 (73%) patients and were distributed as follows: 16 (36%) patients had MMR, 10 (23%) patients had MR4, 8 (18%) patients had MR4.5, 6 (14%) patients had MR5, and 4 (9%) patients were with indetectable transcript (Table 4). The median time to develop a MMR was 13 months (range: 5–84 months). Moreover, we found that an initial optimal response to TKI at 3 months was significantly correlated with the presence of a DMR downstream. Indeed, 26 (90%) patients who developed an initial optimal response at 3 months achieved a subsequent DMR versus 3 (10%) who had an initial warning response at 3 months (*p*=0.00001).

### 3.4. Univariate and Multivariate Analyses of the Impact of Parameters on Optimal Response at 3 Months Achievement

Univariate and multivariate analyses are summarized in Tables 5 and 6. We identified 7 parameters that were significantly correlated with an optimal response to TKI at 3 months. These parameters were subsequently considered as predictive factors of poor response at 3 months and include the following: being male (*p*=0.036, OR: 3.463, 95% CI: 1.186–10.108), a time to diagnosis over 30 days (*p*=0.002, OR: 6.667, 95% CI: 1.874–23.714), an ECOG Performance status 3 and 4 (*p*=0.00004, OR: 13.722, 95% CI: 3.395–55.46), the presence of splenomegaly (*p*=0.0001, OR: 34.133, 95% CI: 4.133–281.924), a high-ELTS risk score (*p*=0.000001, OR: 68.444, 95% CI: 8.052–581.829), the presence of *BCR::ABL1* kinase domain mutation (*p*=0.02, OR: 2.245, 95% CI: 1.543–19.56), and a long delay of CHR over 30 days (*p*=0.000001, OR: 20, 95% CI: 5.384; 74.299).

On the other hand, a warning response to TKI at 3 months (*p*=0.001, OR: 11.118, 95% CI: 2.242–20.186) was significantly correlated with an absence of DMR. In addition, an optimal response at 3 months to TKI was significantly correlated with a DMR. No statistically significant differences were observed with other parameters such as age, hepatomegaly, mean hemoglobin level, mean leukocyte level, platelet count level, and the initial percentage of the *BCR::ABL1* transcript at diagnosis.

## 4. Discussion

The country of Côte d'Ivoire in West Africa has one clinical hematology department located at the Third University Hospital Center, in Yopougon. The department has 10 clinical hematologists for the management of hematology disorders in Côte d'Ivoire. Until recently, many patients suspected of having CML could not benefit from a molecular evaluation because of its high cost. Thanks to the GIPAP, patients can now be diagnosed and also be monitored for their therapeutic response when treated with TKIs. The molecular monitoring of the patients remains the key to the success of the treatment via the evaluation of the molecular response rate. To the best of our knowledge, very few data exist on the assessment of a residual disease and a molecular response to TKI in Africa. Sub-Saharan African countries, including Côte d'Ivoire, have mostly reported studies on the treatment with TKIs but not on their outcomes in patients with CML. We aimed in this study to evaluate early molecular responses (EMRs) to a TKI therapy and to determine predictive factors for poor outcomes and response in patients after imatinib failure. A total of 60 patients diagnosed with CML and treated with TKIs were prospectively monitored through the quantitative evaluation of the *BCR::ABL1* transcript level (by RT-qPCR) at 3 months following initiation of TKI, between May 2018 and June 2023.

### 4.1. Sociodemographic and Clinical and Biological Conditions

The median age of patients enrolled in this study was 45 years (range: 17–75) with a predominance of males (57%). Fleming et al. reported that native African patients less than 40 years old suffered from CML more than any other population in the world. However, this observation was attributed to the age distribution of the African populations rather than to any biological phenomenon [9]. Still, the median age in this study was lower than that reported in Europe and the United States [10]. We observed that 45 (75%) patients had splenomegaly and 8 (13%) had hepatomegaly. The white blood cell (WBC) counts of the patients were higher (298.3 ± 210 × 10^9^/L) than that of the cohort of the IRIS study (median of 17.9 and 20.2, for each respective study). We and others have previously described high WBC counts in sub-Saharan Africans with CML [11, 12]. This observation was linked to a late diagnosis and/or a late presentation of the patients at the hospital and was characterized by a high percentage of splenomegaly and hepatomegaly. The clinical and biological feature of CML in black African patients seems to indicate a more aggressive course of the disease with the high incidence rate of additional chromosomal abnormalities particularly in African CML leading to the poor prognosis of our patients as previously found [11–13].

### 4.2. Hematologic Response

The CHR rate among the patients in the study was 83%. This was achieved within an average time of 2 months (range: 0.6–6 months), a time frame that is slightly higher compared to our previous study [12]. A CHR within 30 days in this study was significantly correlated with an early favorable molecular response. There were no patients who reached a CHR, without developing a favorable molecular response. The CHR rate of 83% is also higher than our previous report of 76% of CHR rate [12]. This higher rate is due to the improvement of the monitoring methods and it outlines a better adherence to the patients and the importance of following up.

### 4.3. Molecular Response at 3 months

According to the guidelines issued by the ELN, the *BCR::ABL1* transcript levels on the IS (*BCR::ABL*1^IS^) at 3 and 6 months are defined as key indicators of the early efficacy of first-line TKI treatment [7]. The first publication on the impact of the EMR, defined as 3-month *BCR::ABL1* transcript < 10% after conversion to the IS, analyzed data from 282 patients newly diagnosed with CP–CML and treated with front-line imatinib. In our study, 48% of patients who achieved an optimal response with TKI therapy after 3 months, represented a much lower proportion than in Europe or the United States [7, 15]. Indeed, in Europe, the German cohort from the previously cited IRIS study where 74.5% of patients achieved a ratio of ≤ 10% at 3 months of treatment with imatinib and the rate of complete cytogenetic response (CCyR) increased to 95% of patients with *BCR::ABL* (IS) ≤ 1.0% by the 12-month landmark. **T**hus, patients with a *BCR::ABL* (IS) > 10% had 84-month EFS rates that were reduced (56%) compared with patients with a *BCR::ABL* (IS) of ≤ 0.1%, *BCR::ABL* (IS) of > 0.1% to ≤ 1.0%, and those with *BCR::ABL* (IS) of > 1% to ≤ 10%, all of whom had an EFS rate > 85% at 84 months. Thus, the OS for patients with a 6-month molecular response of an IS > of 10% was only 68%, compared with > 90% in all other categories of molecular response [15]. Likewise, a study from China reported that 55.7% of patients achieved ≤ 10% with imatinib [16]. On the other hand, the percentage is higher than that reported by Henke et al. [5] in Tanzania, where only 30% of patients achieved a *BCR::ABL1/ABL1* ratio of ≤ 10%. It globally reported that *BCR::ABL1* transcript levels at 3, 6, and 12 months strongly predicted overall survival (OS), progression-free survival (PFS), and event-free survival. Indeed, transcript levels also correlated with the likelihood of achieving a CCyR, a MMR, and a complete molecular response (CMR). [14].

### 4.4. Predictive Factors for Outcome and Molecular Response at 3 months

Many factors have a significant impact on optimal response to TKI at 3 months in this study. This study identified seven independent risk factors that significantly influenced an EMR to TKI. These factors were as follows: (1) being male, (2) having a late diagnosis, (3) having an advanced performance status, (4) the presence of splenomegaly, (5) a high-ELTS stratification score, (6) a kinase domain *BCR::ABL1* mutation, and (7) reaching a CHR after more than 30 days. In addition, we determined that an optimal response to TKI at 3 months was significantly correlated with the development of a DMR. Indeed, 90% of the patients who developed an optimal response at 3 months also achieved a DMR versus 10% of the patients who only showed a warning response to TKI at 3 months (*p*=0.00001). Hughes et al. reported that patients who developed an EMR at 3 months were more likely to achieve a MMR within 2 years compared to patients who had an EMR failure. Reaching an EMR within 3 months correlates well with good prognosis, including improved long-term OS and PFS, and a lower transformation rate to accelerated/blast phases in CP–CML patients [15]. Despite this encouraging information, little is known about the relationship between an EMR and a DMR in CP–CML patients receiving TKI treatment or other factors that contribute to the achievement of a DMR [13, 17].

Furthermore, we determined that baseline factors such as age, hemoglobin level, WBC count, hemoglobin level, platelet, and the initial *BCR::ABL1* transcript levels before treatment were not associated with an optimal response at 3 months. In some studies, high *BCR::ABL1* transcript levels before treatment were reported to be associated with suboptimal responses [16, 18]. In contrast, a recent study demonstrated that the *BCR::ABL1* transcript levels at diagnosis could not predict molecular response and patient survival [19].

## 5. Conclusion

The findings of this study, the first in Côte d'Ivoire, have confirmed the importance of monitoring the level of *BCR::ABL*1^IS^ transcript at 3 months following an imatinib therapy in patients with CP–CML. Our data suggest that a EMR and a DMR can be predicted using a combination of biological characteristics that have a significant impact on an optimal response. An early identification of patients who display a warning response to TKI according to the ELN recommendations may help with the decision for an early administration of an alternative therapy to improve their outcome and the molecular response and also to minimize the risk of the disease progression.

In low-income countries, where the molecular assessment and the monitoring of the disease are not available or not possible, this study indicates that some baseline demographics and biological characteristics can serve as some prognostic indicators for a molecular response. The latter might be more useful for an adapted African prognosis score and for treatment decision-making. Patients from low-resource countries still face massive barriers despite the free treatment, improved diagnostics, and monitoring methods. Bringing treatment to the patients, especially to those in rural areas, might be the key to ensuring a good treatment adherence and a better outcome for sub-Saharan African patients.

## Figures and Tables

**Table 1:** Pretreatment characteristics of the study population of 60 patients with CML.

Variable	Value
Gender, patient count (%)	
Male	34 (57)
Female	26 (43)
Age (yrs) (median range)	45 (17– 75)
Time from diagnostic (month) (median range)	1.5 (0.3–7.5)
ECOG at diagnosis, patient count (%)	
0-1	46 (77)
2-3	14 (23)
Hepatomegaly value (%)	8 (13)
Splenomegaly, patient count (%)	
0 cm	15 (25)
1–9 cm bcma	16 (27)
≥10 cm bcm	29 (48)
Peripheral blood hemoglobin level in g/dL (median range)	9.6 (6–15)
Platelet count (G/L)	588 (144–1653)
Leukocyte count (G/L)	298.370 (10.7–960)
Basophils in PB (%)	1.5 (0–10)
Blasts in PB (%)	1.5 (1–5)
Blasts in BM (%)	5 (2–10)
ELTS risk group, n.(%)	
ELTS low	28 (47)
ELTS intermediate	12 (20)
ELTS high	20 (33)
Duration of imatinib treatment (months)	
< 12	10
12–24	15
> 24	35

*Note:* Cma, centimeters below the costal margin.

**Table 2:** Result of *BCR::ABL1* transcript and mutation screening at diagnosis.

*BCR::ABL1* kinase domain mutation	Number of patients
T315I	3
M351T	1
M244V	1
G255V	1
E255K	1
T315I / M244V	1
E255K	1
G250E / E459K	1
No mutation	20

***BCR *** *:: * ***ABL1* transcript ratio at diagnosis % (60)**	

< 54	18
≥ 54	42

**Table 3:** Response of TKI at 3 months.

Complete hematologic response (CHR)	Patient count (%)
Presence	50 (83)
Absence	10 (17)

**Molecular response to TKI at 3 months**	**n.%**

Optimal: ≤ 10%	29 (48)
Warning:> 10%	31 (52)

**Table 4:** Type of molecular responses at the end of the study.

Molecular responses	Patient count (%)
Presence	44 (73)
Absence	16 (27)

**Type of molecular response**	

MMR	16 (36)
MR4	10 (23)
MR4.5	8 (18)
MR 5	6 (14)
Indetectable	4 (9)

**Table 5:** Univariate analysis of pretreatment biological characteristics and molecular response at 3-month *BCR::ABL1*.

Variable	Optimal ≤ 10% *n* = 29; *n* (%)	Warning > 10% *N* = 31; *n* (%)	*p* value
Age (mean–SD)	41.50 ± 23.335	45.59 ± 15.946	0.40^NS^
Sex			0.036^S^
Male	12(41)	22 (71)	
Female	17(59)	9 (29)	
Time from diagnosis (days)			0.002^S^
≤ 30	25(86)	15 (48)	
> 30	04(14)	16 (52)	
ECOG			0.00004^S^
0-1	26 (90)	12 (39)	
> 2	3 (10)	19 (61)	
Hepatomegaly			0.25^NS^
Presence	2 (7)	6 (19)	
Absence	27 (93)	25 (81)	
Splenomegaly			0.0001^S^
0 (15)	15 (52)	0 (0)	
≥1 (45)	14 (48)	31 (100)	
Hemoglobin (g/dl) mean ± SD	9.62 ± 1.704	8.50 ± 0.989	0.94^NS^
Leukocyte count			
Mean ± SD range (min–max)	178.3 ± 120	245 ± 186	0.76^NS^
Platelet count			
Mean ± SD range (min–max)	417 ± 166	647 ± 359	0.12^NS^
*BCR::ABL1* (%) at diagnosis			0.57^NS^
< 54 (18)	10 (34)	8 (25)	
≥ 54 (42)	19 (65)	23 (74)	
ELTS group, *n* (%)			0.000001^S^
Low (28)	24 (83)	4 (13)	
Intermediate (12)	5 (17)	7 (23)	
High (20)	0 (0)	20 (64)	
Complete hematologic response (days)			0.0000001^S^
≤ 30	24 (83)	6(19)	
> 30	5 (17)	25(81)	
Deep molecular response			0.00001^S^
Presence (28)	26 (90)	2 (6)	
Absence (32)	3 (10)	29 (94)	

Abbreviations: NS, non significant; S, significant.

**Table 6:** Multivariate analysis of pretreatment characteristics and molecular response at 3-month *BCR::ABL1*.

Variable	Optimal ≤ 10% *n* = 29; *n* (%)	Warning > 10% *N* = 31; *n* (%)	*p* value	OR	IC_95%_
Sex					
Male	12 (41)	22(71)	0.036	3.463	[1.186; 10.108]
Female	17 (59)	9(29)	0.036	0.289	[0.099; 0.843]
Time from diagnosis (days)					
≤ 30	25(86)	15(48)	0.002	0.150	[0.042; 0.534]
> 30	04(14)	16(52)	0.002	6.667	[1.874; 23.714]
ECOG					
0-1	26 (90)	12(39)	0.00004	0.073	[0.018; 0.295]
> 2	3(10)	19(61)	0.00004	13.722	[3.395; 55.46]
Splenomegaly					
0 (15)	15(52)	0(0)	0.0001	0.029	[0.004; 0.242]
≥ 1 (45)	14(48)	31(100)	0.0001	34.133	[4.133; 281.924]
ELTS group, *n* (%)					
Low risk (28)	24 (83)	4 (13)	0.000001	0.015	[0.002; 0.124]
Intermediate (12)	5 (17)	7 (23)	0.000001	0.098	[0.052; 0.345]
High (20)	0 (0)	20 (64)	0.000001	57.834	[9.234; 655.487]
Complete hematologic response (days)					
≤ 30	24 (83)	6 (19)	0.0000001	0.050	[0.013; 0.186]
> 30	5 (17)	25 (81)	0.0000001	20.00	[5.384; 74.299]
*BCR::ABL1* mutation					
Presence	0 (0)	10 (45)	0.02	2.245	[1.543–19.56]
Absence	8 (100)	12 (55)	0.02	0.123	[0.087; 0.147]
Deep molecular response					
Presence (28)	26(90)	02(6)	0.00001	0.090	[0.018; 0.446]
Absence (32)	3 (10)	29(94)	0.00001	11.118	[2.242; 20.186]

## Data Availability

The data used to support the findings of this study are available from the corresponding author upon request. The data are not publicly available due to privacy or ethical restrictions.
